# Transmitted Fetal Immune Response in Cases of SARS-CoV-2 Infections during Pregnancy

**DOI:** 10.3390/diagnostics12020245

**Published:** 2022-01-19

**Authors:** Ernesto González-Mesa, Eduardo García-Fuentes, Rafael Carvia-Pontiasec, Ana I. Lavado-Fernández, Celia Cuenca-Marín, María Suárez-Arana, Marta Blasco-Alonso, Blanca Benítez-Lara, Laura Mozas-Benítez, Ana González-Cazorla, Herink Egeberg-Neverdal, Jesús S. Jiménez-López

**Affiliations:** 1 Biochemistry and Inmunology Department, Malaga Biomedical Research Institute-IBIMA, University of Málaga, Surgical Specialities, 29010 Málaga, Spain; blancabenl15@gmail.com (B.B.-L.); mozasbenitezlaura@gmail.com (L.M.-B.); anagonzalezcazorla2@gmail.com (A.G.-C.); Henrikegebergneverdal@gmail.com (H.E.-N.); jesuss.jimenez.sspa@juntadeandalucia.es (J.S.J.-L.); 2 Obstetrics and Gynecology Department, Málaga Regional Maternity Hospital (SSPA), 29010 Málaga, Spain; cecuman2@yahoo.es (C.C.-M.); dramariasuarez@gmail.com (M.S.-A.); martablascoalonso@gmail.com (M.B.-A.); 3 Digestive System Clinical Management Unit, Malaga Biomedical Research Institute-IBIMA, Virgen de la Victoria University Hospital, 29010 Málaga, Spain; edugf1@gmail.com; 4 Provincial Unit of Pathological Anatomy of Malaga, Regional University Hospital of Malaga, SSPA, 29010 Málaga, Spain; resteban.carvia.sspa@juntadeandalucia.es (R.C.-P.); anabelap86@gmail.com (A.I.L.-F.)

**Keywords:** COVID-19 and pregnancy, SARS-CoV-2 infection, fetal lymphocyte subsets, fetal immune system

## Abstract

(1) Background: Little is known about the effects of SARS-CoV-2 on the placenta, and whether the maternal inflammatory response is transmitted vertically. This research aims to provide information about the effects of SARS-CoV-2 infection on maternal and fetal immunity. (2) Methods: We have studied placental changes and humoral and cellular immunity in maternal and umbilical cord blood (UCB) samples from a group of pregnant women delivering after the diagnosis of SARS-CoV-2 infection during pregnancy. IgG and IgM SARS-CoV-2 antibodies, Interleukin 1b (IL1b), Interleukin 6 (IL6), and gamma-Interferon (IFN-γ), have been studied in the UCB samples. Lymphocyte subsets were studied according to CD3, CD8, CD4, CD34, and invariant natural Killer T cells (iNKT) markers. We used in situ hybridization techniques for the detection of viral RNA in placentas. (3) Results: During the study period, 79 pregnant women and their corresponding newborns were recruited. The main gestational age at the time of delivery was 39.1 weeks (SD 1.3). We did not find traces of the SARS-CoV-2 virus RNA in any of the analyzed placental samples. Detectable concentrations of IgG anti-SARS-CoV-2 antibodies, IL1b, IL6, and IFN-γ, in UCB were found in all cases, but IgM antibodies anti-ARS-CoV-2 were systematically undetectable. We found significant correlations between fetal CD3+ mononuclear cells and UCB IgG concentrations. We also found significant correlations between UCB IgG concentrations and fetal CD3+/CD4+, as well as CD3+/CD8+ T cells subsets. We also discovered that fetal CD3+/CD8+ cell counts were significantly higher in those cases with placental infarctions. (4) Conclusion: we have not verified the placental transfer of SARS-CoV-2. However, we have discovered that a significant immune response is being transmitted to the fetus in cases of SARS-CoV-2 maternal infection.

## 1. Introduction

Some previous studies revealed that pregnant women with different viral respiratory diseases were at high risk of developing obstetric complications and adverse perinatal outcomes related to changes in the immune response [1,2,3]. Most pregnant women who become infected by SARS-CoV-2 virus are generally asymptomatic or mildly symptomatic [4], but it is known that they are at increased risk of complications, severe morbidity, or mortality compared to the general population, as seen in other coronavirus infections [1,5,6,7], regardless of gestational age [8]. Pregnant women presenting with pneumonia or acute respiratory distress syndrome associated with COVID-19 have poorer obstetric and perinatal outcomes and a higher number of preterm cesarean sections [9]. Additionally, COVID-19 infection can cause adverse effects during pregnancy such as restricted fetal growth, preterm delivery, and perinatal mortality [1,5,6,7,10]. However, the exact mechanisms by which the infection produces these outcomes are not well known. While some studies assure that maternal hypoxemia caused by respiratory infection can be the cause of fetal hypoxia and the consequent complications of childbirth, others suggest that a direct effect of the virus on the placenta could be the cause of fetal morbidity and mortality [11,12,13,14]. COVID-19 and preeclampsia are strongly associated [15,16], and both have been reported to have additive negative effects during the pregnancy [16]. Previous vascular conditions and COVID-19 inflammatory changes have been suggested as the main responsible factors [15,16].

During pregnancy, many physiological changes occur, which have a significant impact on the immune, respiratory, cardiovascular, and blood clotting systems. The immune system adapts during pregnancy [17] to allow the growth of a semi-allogenic fetus, resulting in a probably altered immune response to infections. Pregnancy involves two immune challenges: establishing and maintaining tolerance to the fetus and, on the other hand, maintaining the ability to protect against microorganisms. In the first trimester, a pro-inflammatory state dictates necessary changes for the implantation of the zygote and development of the placenta; in the second trimester, it changes to an anti-inflammatory state to allow the fetus to grow; and in the third trimester, a pro-inflammatory state is reached again to prepare for childbirth [18,19].

ACE-2 receptors are important in a SARS-CoV-2 virus tissue invasion, and these receptors have been described in placental tissue from all trimesters [20,21]. However, little is known of the effects of SARS-CoV-2 on the placenta [11,12,13,14]. Moreover, the possible transplacental transfer of the virus has not been fully clarified [22,23], although vertical transmission has been reported in some cases of COVID-19, complicating gestation [20,24,25]. Additionally, it is not clear whether the maternal inflammatory response to infection can be transmitted to the fetus through the placental pathway, inducing changes in the fetal immune system [26].

This research report provides information about the effects of SARS-CoV-2 infection on maternal and fetal immunity and about the correlations between them and placental changes. The objective of this work was to study if the effects of maternal inflammatory signals could be recognized in fetal immunity system.

## 2. Materials and Methods

This research was an observational and prospective study, carried out on pregnant women who were suffering at the moment of delivery or who had suffered from SARS-CoV-2 infection throughout their pregnancy. The target population included any laboring woman with a positive SARS-CoV-2 PCR test either at hospital admission or at any time during the pregnancy. The study was performed in the Regional University Hospital of Málaga, within the Andalusian public health system (SSPA). Recruitment was carried out through consecutive sampling during the six months in which the study was active (from November 2020 to May 2021). The only exclusion criterion was the inability to give informed consent in the absence of a legal representative. All women invited to participate gave their consent.

### 2.1. Ethical Aspects

This study was carried out with respect for the fundamental rights and ethical postulates that affect biomedical research on human beings, following the international recommendations contained in the Declaration of Helsinki and its subsequent revisions. Likewise, the national recommendations have been followed in accordance with the national Biomedical Research Law 14/2007. The local ethical committee of Málaga authorized the research.

### 2.2. Procedures

Before the beginning of the study, sample collection kits were prepared, in which 3 tubes with EDTA were included for the cord blood samples, as well as a reservoir for the placentas with their corresponding anonymized identification by means of a code that allowed maintaining confidentiality. The samples were collected by midwives or obstetricians who attended the delivery. All the information related to the samples, as well as all the personal and clinical data associated with the samples, were considered confidential and were treated in accordance with the provisions of the General Data Protection Spanish regulations, Organic Law 3/2018 of 5 December regarding the protection of personal data and guarantee of digital rights, and Law 14/2007 of July 3 on biomedical research.

During the study period, the clinical protocols changed according to the regulations of the health authorities, although the PCR test in the nasopharyngeal exudate for the detection of the virus was systematically performed on all pregnant women who were admitted to hospital. Likewise, the clinical history recorded the possible incidents that occurred during pregnancy, including the positivity of the diagnostic tests for SARS-CoV-2 infection and the different clinical forms of presentation of COVID-19.

Once the participants’ informed consent was obtained, at the time of delivery, the blood samples were collected and kept in a refrigerator at 15 degrees Celsius until the Biobank staff collected and processed them for their conservation at minus 80 °C. The samples were managed by the Biobank Provincial Málaga-IBIMA-SSPA, following standardized work protocols. The Malaga-IBIMA-SSPA Provincial Biobank is part of the Biobank of the Andalusian Public Health System (BSSPA) and the National Biobank Platform (exp. PT17/0015/0041). Blood samples were collected from the mother at the time of labor and from the umbilical cord immediately after birth. Placentas were stored at minus 30 °C until they were examined.

### 2.3. Analysis

The humoral immunity mediators IgG and IgM anti-SARS-CoV-2 antibodies, IL1b, IL6, and IFN-γ, were tested using commercial ELISA assays [27]. They are high sensibility and sensitivity tests, that have been tested for cross reactivity. In Table 1, the main characteristics of these tests are shown.

The placental tissue samples were processed for histological study. Finally, to detect viral RNA in placental tissue, we used in situ hybridization analysis. Commercial BOND RNAscope^®^ Brown Detection [28] allows the visualization of target RNA molecules through chromogenic conversion.

## 3. Results

During the study period, 79 pregnant women and their corresponding newborns were recruited. All cases were simple pregnancies. The mean age of the participants was 31.9 years (SD 6.3). None of the participants had severe pneumonia that required admission to the ICU, and most of them (72.1%) suffered from infection during the third trimester (mean gestational age at the time of infection 31.2 weeks (SD 8.3). The mean gestational age at the time of delivery was 39.1 weeks (SD 1.3). The prematurity rate in the participants was 5.1%, and these cases consisted of four deliveries at week 36. In most cases (64.6%), the delivery started spontaneously, but the induction of labor was necessary in 26.6% of the cases. One of the cases was an elective caesarean section.

The reasons for induction were: 11 cases of premature rupture of the membranes (13.9%), 7 cases of fetal distress (8.9%), and 4 prolonged pregnancies (over week 41). Regarding the newborns, 46.9% were female. The mean weight at birth was 3246 g (SD 438), so that only two newborns (2.6%) had a weight under 2500 g, and four (5.1%) had weights above 4000 g. The Apgar test scores were higher than 7 at the first and fifth minute in all cases, except for one of the newborns, who did not present any complications and could be discharged. Only three newborns required admission to the neonatal intensive care unit: one due to neonatal sepsis in a case of spontaneous delivery at 36 weeks in which the mother had been infected by SARS-CoV-2 in the second trimester with a mild form of the disease; another due to respiratory distress syndrome in a case of spontaneous delivery at 36 weeks with a positive SARS-CoV-2 test at admission for delivery; and a full-term newborn with an atrial septal defect. All three cases could be discharged in a healthy condition. The rest of the newborns did not present any complications until hospital discharge. 

**Figure 1:**
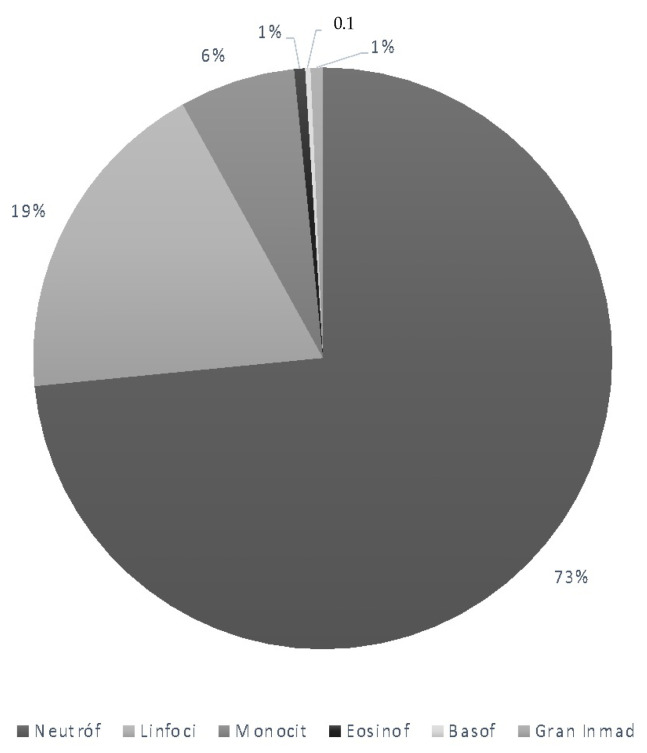
Maternal leucocyte subtypes.

The analytical values corresponding to the hematological study in maternal blood are shown in the following Table 2 and Figure 1.

**Figure 2:**
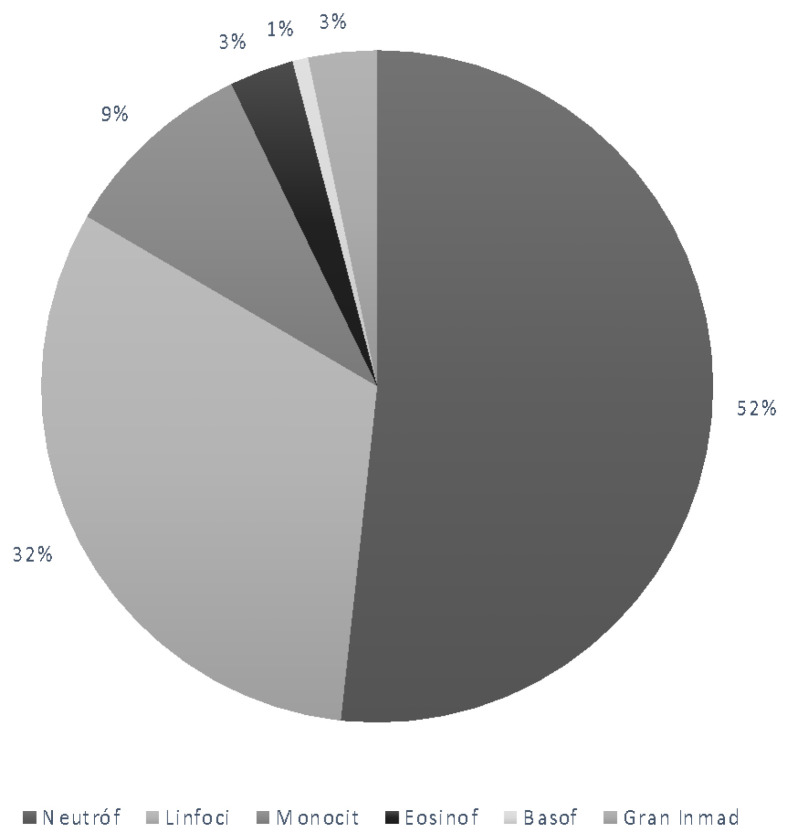
Umbilical cord blood samples. White blood cell subtypes.

The hematological values in umbilical cord blood are shown in Table 3 and Figure 2.

The mean values and corresponding standard deviations of IgG anti-SARS-CoV-2, IgM anti-SARS-CoV-2, IL6, IL1b, and IFN-γ in umbilical cord blood are shown in the Table 4.

We observed statistically significant positive correlations between the time of evolution of the infection and the concentrations of IgG antibodies (Pearson’s coefficient r = 0.26, *p* < 0.02) and IFN-γ (Pearson’s coefficient r = 0.27, *p* < 0.01) in the UCB of the participants. Likewise, the concentrations of IL6 and IL1b were significantly correlated (Table 5).

Table 6 shows the correlation of antibodies and elements of humoral immunity with the maternal blood cells. A significant correlation is observed between IL1b levels and the number of circulating eosinophils.

The correlations between the level of antibodies and other mediators of humoral immunity, and the counts of fetal blood cells are shown in Table 7.

We have observed that cases with very high IgG anti-SARS-CoV-2 concentrations (over the 75th percentile) were associated with higher fetal neutrophil counts and lower lymphocyte counts. Finally, in cases where the IFN-γ concentrations were high (over the 75th percentile), the fetal neutrophil count was also higher. Table 8 shows the average values.

**Figure 3:**
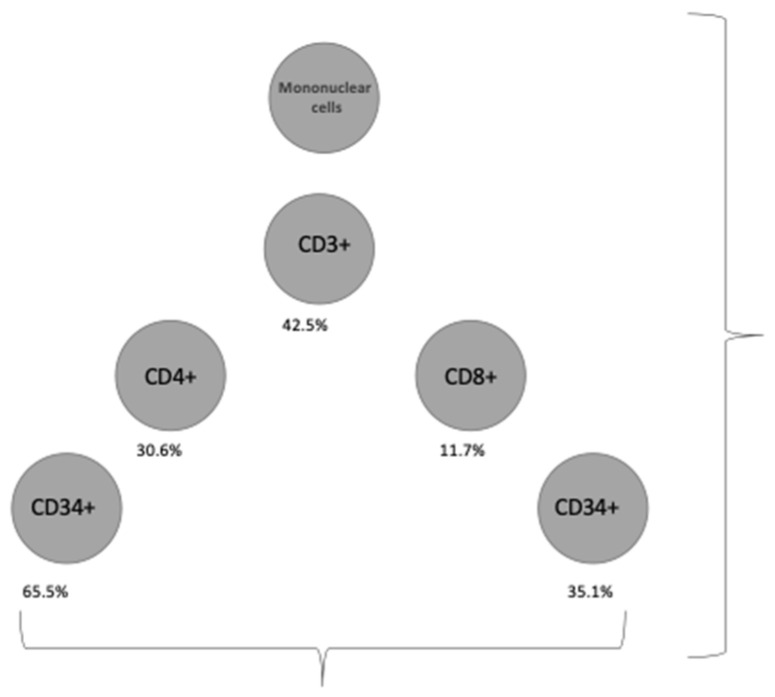
Cellular subsets.

Regarding cellular immunity, Table 9 and Figure 3 show the proportion of cell subpopulations represented over the total mononuclear cells circulating in UCB. Table 9 shows the information on the subpopulations of T lymphocytes analyzed.

Table 10 shows the correlations between lymphocyte subpopulations in UCB and maternal hematological parameters. We have observed a negative and statistically significant correlation of the number of CD4+ T lymphocytes and the number of maternal neutrophils. Similarly, we have observed a negative correlation between the number of CD4+ and CD34+ T lymphocytes, and the number of circulating mature maternal granulocytes.

Table 11 shows the correlations between lymphocyte subpopulations in UCB and neonatal hematological parameters. We observed a negative and statistically significant correlation between the proportion of T lymphocytes and immature granulocytes. We also observed significant correlations between the proportion of iNKT+ cells and the number of neutrophils, lymphocytes, monocytes, eosinophils, and basophils circulating in UCB.

UCB circulating IgG levels show significant correlation with the proportion of T lymphocytes in the mononuclear cell population and, specifically, with the CD4+ and CD8+ lymphocyte subpopulations. The CD8+/CD34+cell subpopulation and the iNKT subpopulation show significant correlation with IL1b concentrations (Table 12).

We observed a negative correlation between fetal weight and the proportion of CD3+ lymphocytes (r = −0.33, *p* = 0.05) and CD4+ lymphocytes (r = −0.33, *p* = 0.037); however, we did not find any relationship between the different cell subpopulations and gestational age at the time of delivery, maternal age, type of delivery, form of delivery onset, or the trimester of infection.

We found a higher proportion of CD3+/CD8+/CD34+ lymphocytes in cases in which the placenta showed infarction areas (mean 32.8, SD 14.05, versus 44.27 SD 12.99, *p* = 0.044). On the other hand, the observed proportion of CD3+/CD8+ lymphocytes was significantly higher in cases in which SARS-CoV-2 infection had evolved over two weeks, compared to cases in which the diagnosis was in the 24 h prior to delivery (mean 9.79 SD = 3.0, compared to 13.73. SD = 4.9; F = 7.66; *p* = 0.009).

### Placental Study

We found no RNA of the virus in the placental tissue analyzed until this time, nor clear evidence of the transplacental passage of the virus. However, we observed the existence of placental changes that could be related to the effects of SARS-CoV-2 infection, especially of the ischemic type, pointing to the existence of vascular accidents in placental microcirculation.

## 4. Discussion

In this research report, we have studied placental changes and humoral and cellular immunity in maternal and umbilical cord blood (UCB) samples from a group of pregnant women delivering after a diagnosis of SARS-CoV-2 infection during pregnancy.

Regarding fetal transmission of SARS-CoV-2, our in situ hybridization (RNA scope) analysis failed in the detection of RNA traces. Moreover, newborns had negative PCR analyses in all cases. Although the existence of receptors in trophoblastic tissue has been described [24,29,30], and it is known that trophoblastic cells can produce type II transmembrane serine-protease (the main mediator of the entry of the virus into cells), our results show that, at least in our samples, the placenta has not been a specific target organ for the virus.

It should be considered that the expression of ACE-II and type II transmembrane serine-protease receptors in third-trimester placentas might be less relevant [31], making vertical transmission pathway unimportant and reserved for cases in which some gestational conditions related to the renin–angiotensin axis could modify the expression of ACE-II receptors or facilitate other routes of access to the fetal compartment. Our sample of pregnant women in the third trimester, who, in general, had no special risk factors, demonstrated results that would support this hypothesis.

On the other hand, pathological analysis showed signs of placental vascular mal-perfusion (mainly placental infarctions and retroplacental hemorrhages) in 37.2% of cases, similar to those described in previous studies [32].

Some previous studies showed a lack of transplacental passage of pro-inflammatory cytokines [33,34], so the finding of detectable concentrations of IL-1b, IL-6, and IFN-γ are most likely related to the triggering of a certain fetal inflammatory response. In fact, IFN-γ concentrations correlated with WBC count in the newborn, indicating the existence of neonatal immune reactivity. The placental transfer of immunoglobulin has been previously reported [34,35], especially IgG, and to a lesser extent, IgM [35]. In the sample that we have studied, we detected antibodies of the IgG type in the umbilical cord blood in increasing concentrations as the time of the evolution of the disease increased.

At the time of writing this research report, we studied lymphocyte subsets from 44 of the recruited cases. We observed that 42.5% of mononuclear cells express CD3, corresponding to T lymphocytes. The ratio of CD4/CD8 lymphocytes was 2.8 (SD 1.19). Several findings support our research hypothesis regarding the existence of a cellular immunity response transmitted to the fetus. Firstly, the observed relationship between the concentration of anti-SARS-CoV-2 IgG antibody and the cellular count of CD3+ lymphocytes points towards the existence of a fetal cellular response, with an increase in the production of T lymphocytes. Moreover, within CD3+ lymphocytes, an increase in CD4+ and CD8+ lymphocytes counts has been observed, with a predominance of CD8, as anti-SARS-CoV-2 IgG levels increase. These findings are in line with previous reports about the increased capacity of fetal T-cell subsets to cytokine stimulation [36]. Finally, pathological analysis of placentas showed that signs of poor perfusion (placental infarctions) were associated with higher levels of CD3+/CD8+ lymphocytes.

One of the most important limitations of the present study is the absence of a control group. It is, therefore, a correlational study from which it is not possible to obtain causal relationships. However, though these results should be considered preliminary and need an independent confirmation and validation with a control group, the findings are strong enough to support the hypothesis of fetal transmission of the maternal inflammatory response after SARS-CoV-2 infection complicating the pregnancy.

## 5. Conclusions

We obtained data on the status of hematological and immunological parameters in maternal and umbilical cord blood in cases of SARS-CoV-2 infection during pregnancy. We could not find any traces of SARS-CoV-2 in placental samples. However, the relationship between fetal cellularity and the concentrations of humoral/cellular immunity components points to the existence of vertical transmission of an inflammatory response, observing changes in the concentration of inflammation mediators in fetal blood.

## Figures and Tables

**Table 1:** Main properties of the tests.

	Minimum Detectable Dose	Intra-Assay Variation Coefficient	Inter-Assay Variation Coefficient	Tested Absence of Cross-Reactivity
RayBio^®^ Human IL-1 beta ELISA Kit (RayBio^®^ ref. ELH-IL1b)	0.3 pg/mL	<10%	<12%	IL-1α. IL-1β. IL-2. IL-4. IL-8. IL-10. IL-12. IL-13. IFNγ and TNFα
Human IL-6 High Sensitivity ELISA Kit (Diaclone. ref. 950.035.096)	0.81 pg/mL	4.4%	9.1%	IL-1α, IL-1β, IL-2, IL-4, IL-8, IL-10, IL-12, IL-12, IFNγ y TNFα
Human IFNγ High Sensitivity ELISA Kit (Diaclone. ref. 850.900.096)	0.69 pg/ml	3.9%	8.6%	IL-1α. IL-2. IL-8. IL-12p40. TNFα. CD95/Fas. TRAIL. ICAM-1. gp130 and GM-CSF
Human SARS-CoV-2 Spike Protein S1 IgM (MyBioSource. ref. MBS2614311)	1.2 U/mL	8%	12%	IgM analogues
COVID-19 (SARS-CoV-2) quantitative IgG ELISA (Demeditec. ref. DECOV1901Q)	3.6 AU/mL	5.3%	9.9%	IgG analogues

**Table 2:** Maternal blood cell count.

		Hb	WBC	Platelets	Neutrophils	Lymphocytes	Monocytes	Eosinophils	Basophils	Granulocytes Immature (%)
Mean		11.60	10.67	231.08	8.06	1.83	0.67	0.06	0.03	0.79
Std. Deviation		1.250	3.44	67.96	3.28	0.57	0.24	0.08	0.01	0.83
Minimum		7.90	4.79	99.00	3.18	0.77	0.33	0.00	0.01	0.03
Maximum		14.00	20.66	408.00	18.77	3.79	1.71	0.50	0.09	6.00
Percentiles	25	10.87	8.08	182.00	5.68	1.43	0.50	0.02	0.02	0.4
	50	11.60	9.79	234.00	7.06	1.84	0.62	0.03	0.03	0.60
	75	12.42	12.54	268.75	9.76	2.19	0.76	0.08	0.04	0.95

**Table 3:** Umbilical cord blood samples. Cell counts.

	Hb	WBC	Plate- Lets	Neutrophils	Lymphocytes	Monocytes	Eosinophils	Basophils	Granulocyte Immature (%)	Erythroblasts
Mean	15.91	14.58	305.23	7.89	4.96	1.50	0.50	0.14	3.40	0.88
Std. Deviation	1.67	4.04	70.55	3.06	2.46	0.71	0.38	0.20	2.44	1.45
Minimum	12.80	8.20	34.00	2.56	2.78	0.69	0.00	0.01	0.08	0.00
Maximum	22.80	25.55	470.00	18.00	20.30	5.30	2.50	1.50	9.60	9.10
25th Percentile	14.77	11.38	269.00	5.83	3.80	1.05	0.28	0.06	1.10	0.20
50th Percentile	15.75	14.24	300.00	7.14	4.36	1.41	0.42	0.10	3.00	0.40
75th Percentile	16.80	18.11	347.00	8.94	5.69	1.75	0.61	.014	5.00	0.90

**Table 4:** Umbilical cord blood samples. Cytokines concentration.

		Ab IgM (U/mL)	Ab IgG (U/mL)	IL-6 (pg/mL)	IL1B (pg/mL)	IFN-γ (pg/mL)
Mean		0.17	4227.91	11.53	0.58	7.02
Std. Deviation		0.36	5817.53	20.46	0.32	4.97
Minimum		0.00	108.2	2.1	0.3	0.7
Maximum		1.55	28348.6	109.1	1.9	18.8
Percentiles	25	0.00	246.81	3.18	0.43	1.56
	50	0.00	1798.70	4.21	0.47	6.85
	75	0.15	5871.07	7.29	0.55	10.64

**Table 5:** Umbilical cord blood samples. Correlations between cytokines concentrations. Pearson’s coefficient. Pearson’s significant correlation coefficients have been highlighted.

	Time of Evolution	Ab IgG (U/mL)	IL-6 (pg/mL)	IL1B (pg/mL)	IFN-γ (pg/mL)
Ab IgG (U/mL)	0.301 **	1			
IL-6 (pg/mL)	0.053	0.002	1		
IL1B (pg/mL)	−0.138	0.030	0.397 **	1	
IFNg (pg/mL)	0.280 **	0.064	−0.013	−0.083	1

** Significant (*p* < 0.01).

**Table 6:** Correlations between cytokines concentrations and maternal blood cells count. Pearson’s significant correlation coefficients have been highlighted.

	Hb	WBC	Platelets	Neutrophils	Lymphocytes	Monocytes	Eosinophils	Basophiles	Granulocytes Immature
Ab IgG (U/mL)	−0.17	0.094	0.052	0.121	−0.130	0.014	−0.039	−0.011	−0.056
IL-6 (pg/mL)	−0.08	0.037	−0.084	0.055	−0.090	−0.001	−0.037	0.109	0.071
IL1B (pg/mL)	0.00	0.017	0.051	−0.002	0.095	0.027	0.336 *	0.046	−0.022
IFN-γ (pg/mL)	0.14	0.080	0.072	0.121	−0.170	−0.057	−0.155	0.167	0.127

* Significant (*p* < 0.05).

**Table 7:** Correlations between cytokines concentrations and umbilical cord blood cells count. Pearson’s coefficient. Pearson’s significant correlation coefficients have been highlighted.

	Hb	WBC	Platelets	Neutrophils	Lymphocytes	Monocytes	Eosinophils	Basophils	Granulocytes Immature (%)	Erythroblasts
Ab IgG (U/mL)	0.002	−0.014	0.078	0.093	0.001	0.011	−0.168	0.013	0.028	0.126
IL-6 (pg/mL)	−0.111	−0.085	0.155	0.167	0.006	0.094	−0.103	−0.027	0.146	−0.111
IL1B (pg/mL)	−0.005	−0.058	0.009	0.002	−0.181	−0.125	−0.144	−0.205	−0.066	0.111
IFN-γ (pg/mL)	0.111	0.020	0.261 *	0.308	−0.106	0.070	0.169	−0.012	0.332 *^	−0.029

* Significant (*p* < 0.05).

**Table 8:** Main values in neutrophils and lymphocytes counts according to IgG concentration (*p* < 0.005).

	Neutrophils	Lymphocytes
IgG		
<75th Percentile	7.88 (3.1)	4.96 (2.6)
>75th Percentile	7.94 (2.93)	4.95 (1.6)
Interferon		-
<75th Percentile	7.68 (2.76)	-
>75th Percentile	8.82 (4.18)	-

**Table 9:** Lymphocyte subset proportions.

Subsets	Mean	Median	Std Dev	*p* 25	*p* 50	*p* 75
% CD3+	42.52	40.15	12.948	31.55	40.15	53.675
% CD3+INKT+	1.695	1	1.7929	0.3	1	2.6
% CD3+INKT+CD34+	88.573	89.05	6.779	84.675	89.05	92.1
% CD3+CD4+	30.69	30.7	11.154	19.025	30.7	39.875
% CD3+CD4+CD34+	65.115	64.55	10.842	54.825	64.55	75.925
CD3+CD8+	11.708	11.1	4.497	8.225	11.1	14.375
% CD3+CD8+CD34+	35.132	32.25	14.446	23.8	32.25	45.7
Ratio CD4+/CD8+	2.874	2.67	1.198	2.199	2.674	3.555
Ratio CD4+CD34+/CD8+CD34+	2.089	2.006	0.667	1.59	2.00	2.44

**Table 10:** Correlations between umbilical cord blood lymphocyte subsets and maternal blood cells count. Pearson’s significant correlation coefficients have been highlighted.

	CD3+	CD3+ INKT+	CD3+INKT+ CD34+	CD3+ CD4+	CD3+CD4+ CD34+	CD3+ CD8+	CD3+CD8+ CD34+
Hb	0.140	0.044	0.014	0.165	0.065	0.012	−0.021
WBC	−0.310	0.120	0.069	−0.426	0.036	0.132	0.080
Platelets	−0.362	0.135	−0.270	−0.275	−0.237	−0.310	0.030
Neutrophils	−0.329	0.094	0.118	−0.452 *	0.031	0.143	0.070
Lymphocytes	−0.009	0.076	−0.255	0.010	−0.108	−0.066	−0.033
Monocytes	−0.088	0.257	0.088	−0.144	0.290	0.078	0.229
Eosinophils	−0.034	0.089	−0.138	−0.070	0.184	0.075	0.237
Basophils	−0.169	0.013	−0.386	−0.211	−0.168	0.041	−0.067
Granulocytes	−0.189	−0.253	−0.385	−0.176	−0.352 *	−0.094	−0.296

* Significant (*p* < 0.05).

**Table 11:** Correlations between umbilical cord blood lymphocyte subsets and neonatal blood cells count. Pearson’s significant correlation coefficients have been highlighted.

	CD3+	CD3+ INKT+	CD3+INKT+ CD34+	CD3+ CD4+	CD3+CD4+ CD34+	CD3+ CD8+	CD3+CD8+ CD34+
Hb	0.048	−0.069	0.299	0.096	0.199	−0.122	0.088
WBC	−0.117	0.033	−0.068	−0.158	−0.131	0.038	−0.004
Platelets	−0.268	0.335	−0.101	−0.199	0.126	−0.281	0.107
Neutrophils	−0.247	0.445 *	−0.214	−0.186	0.084	−0.229	0.201
Lymphocytes	−0.251	0.581 **	−0.238	−0.188	0.246	−0.238	0.287
Monocytes	−0.237	0.568 **	−0.225	−0.178	0.219	−0.221	0.303
Eosinophils	−0.108	0.437 *	−0.059	−0.085	0.322	−0.086	0.307
Basophils	−0.289	0.607 **	−0.266	−0.238	0.150	−0.211	0.311
Granulocytes	−0.315	0.272	0.123	−0.280	0.178	−0.239	0.152
Erythroblasts	−0.381 *	0.210	−0.025	−0.301	−0.171	−0.338	−0.039

* Significant (*p* < 0.05); ** significant (*p* < 0.01).

**Table 12:** Correlations between umbilical cord blood lymphocyte subsets and umbilical cord blood cytokines concentrations. Pearson’s significant correlation coefficients have been highlighted.

	CD3+	CD3+ INKT+	CD3+INKT+ CD34+	CD3+ CD4+	CD3+CD4+ CD34+	CD3+ CD8+	CD3+CD8+ CD34+
Ab IgG (U/mL)	0.434 **	0.079	0.069	0.316 *	0.217	0.481 **	0.156
IL-6 (pg/mL)	−0.047	−0.062	−0.028	−0.074	−0.128	0.047	−0.139
IL1B (pg/mL)	−0.264	0.460 **	−0.186	−0.218	0.105	−0.213	0.333 *

* Significant (*p* < 0.05); ** significant (*p* < 0.01).

## Data Availability

All presented data are available under reasonable request to corresponding author.
